# On the Efficacy of Small Doses of Morphia in Certain Chest Diseases

**Published:** 1856-07

**Authors:** 


### ECLECTIC DEPARTMENT,

### AND SPIRIT OF THE MEDICAL PERIODICAL PRESS.

---

*On the efficacy of Small Doses of Morphia in certain Chest Diseases.*—Dr. Edward Smith read before the Medical Society of London, January 12, 1856, a paper on this subject. The dose referred to by the author was from the sixty-fourth to the twenty-fourth of a grain for an infant or young child, and from the twentieth to the twelfth of a grain for an adult, repeated from three to six times in the twenty-four hours. The principle involved was the removal of excessive cough in those cases in which the cough resulted from nervous irritability of the structures of the air-passages, and thereby to prevent various ill effects which would follow from its continuance. He did not regard the cough as a symptom merely, but as the result of irritation, and it was by the removal of the irritation that the cough was to be prevented. He showed that the effect of minute and often repeated doses of morphia was far more efficacious than occasional and larger doses, and that the sensorium remained quite unaffected, and the bowels nearly so, under its influence, and therefore that no disturbance to the general system was produced by its administration. The author selected three diseases in illustration of the merits of the remedy, viz: whooping-cough, the ordinary and spasmodic form of chronic bronchitis, and phthisis. Whooping-cough he regarded as essentially a disease of the nervous system, quite apart from inflammation, and considered the principle of treatment to be the removal of the spasm, so that the cough might be reduced to the harmless state of common cough. He believed the secretion to be chiefly due to the violence of the cough, and consequently that the aim should not be to increase the secretion by expectorants, but to stop the cough, and allow the secretion to cease. He was also of opinion that the congestion of the lungs in more advanced cases, which often leads to other complications, is chiefly due to the spasmodic cough. Thus he inferred that the prevention of all serious and fatal complications. The treatment he recommended was first to remove all sources of irritation; to give nutritive food in small quantities and very frequently; to expose the patient to cool pure air, and in general to place it in the best sanitary conditions, and then to administer the morphia. The system adopted in giving the morphia was to begin with a very small dose (say the sixty-fourth of a grain to an infant four months old) and, if need be, to rapidly increase it until the slightest drowsiness was perceptible, and then to regard that effect as the measure of the dose, and to continue the dose and effect until the spasm subsided. He particularly insisted upon the fact that any dose less than sufficient to affect the sensorium in the slightest degree was sufficient for the immediate cure of the disease, and therefore that the art in the administration was to produce and maintain that effect in the quickest way, and with the smallest dose. After giving the sixty-fourth of a grain for three or four doses, without any drowsiness, he would then increase it to the forty-eighth of a grain for three or four more doses, and again, if need be, to the thirty-eighth and thirty-second of a grain until the slightest drowsiness appeared. Thus the proper dose would soon be ascertained, and within one or two days the spasm would be materially lessened. He stated that within four, and commonly within ten days, he had cured severe cases so far as to reduce the spasmodic to a common cough, and to prevent the recurrence of any complication. The points he especially urged were, the rapid increase of the dose, to produce the desired effect, and the careful maintenance of the effect by regulating the dose. He insisted upon the necessity of using a graduated measure in the administration of the remedy.

*Chronic Bronchitis.*—The attacks of this disease, as commonly seen in the *habitués* of hospitals, he believed not to be inflammatory but nervous or spasmodic, both in the cough and in the dyspnœ, and also even in the sense of constriction of the chest; and that the aim should not be to increase the secretion, but simply and purely to remove the nervous condition on which the cough, secretion, dyspnœa, and tightness depended. The cough he believed to be much more than needful to remove detached phlegm, and by its continuance to excite the secretion, and he inferred that when relief was obtained with the increase of secretion the former was not due to the latter, but both resulted from exhaustion of the nervous irritability by lapse of time. He therefore commonly banished expectorants, and administered small doses of morphia. The dose was usually the sixteenth of a grain, repeated three or four times a day. Very speedily the cough was relieved, and then the dyspnœa, and at the same time or subsequently the sense of tightness. He maintained that the same remedy which first relieved the cough only, would, by continuance or increase, relieve the dyspnœa also. As a preliminary step, all sources of irritation were attended to, especially the liver, stomach, and bowels. The croton oil liniment was efficacious only on the principle laid down by the author, and not upon the relief of any supposed inflammatory action.

*Phthisis.*—The exhibition of small doses of morphia in phthisis, was not with a view to cure that disease, but to lessen or prevent the occurrence of certain important complications, which the author believed to be in great part due to the cough; such hæmoptysis, congestion or inflammatory action, vomiting after food, increase of night perspiration, disturbance of sleep, and the increase of the general irritability of the system. He was decidedly of opinion, that in all stages of the disease the cough is commonly greater than is required upon the principles of relieving the system, and therefore, and because it leads to further evils, it ought to be arrested. He especially referred to two periods. 1st. In the early stage, when there is scarcely any secretion, and when the patient voluntarily adds to the cough, to remove a little phlegm, which he believes to be its cause. He considered it essential to disabuse the patient's mind of this error, and by that means, and by the aid of morphia, to arrest the cough. 2d. He had ascertained that the vomiting of the food after meals was not usually due to disorder of the digestive organs, but to cough accompanying an irritable state of the general system, and a state of repletion of the stomach, and he had commonly prevented it by reducing the quantity of food at each mal (giving it more frequently,) and by administering the sixteenth of a grain of morphia immediately before or after a meal, as well as at the usual intervals during the day. A more tranquil sleep and less severe nightsweats resulted from the small dose of morphia repeated once or twice in the night than when one large dose was given.

In some of the above conditions the author considered the cough to have the importance not merely of a symptom but of a substantive disease, and as such to require especial attention in the treatment; but as it was oftentimes associated with constitutional ailments, he frequently combined the morphia with vegetable bitters, and with the sesquichloride of iron.

He (Dr. Edward Smith) preferred morphia to other narcotics, because its strength is more uniform than that of vegetable extracts, and more permanent than that of hydrocyanic acid; and upon the whole it was easily capable of minute subdivision and increase in its dose, and was less dangerous in its administration.—*Lancet*, Jan. 26, 1856.

---

*Paper containing Arsenic.*—In these days, when the toxicologist is so frequently puzzled to know how the poison which his skill has detected has got into the organismus, it may not be unimportant to know that a great quantity of blotting-paper, which is frequently used for filtering, contains arsenic in considerable quantity. Vohl has found, in a grey kind of blotting-paper, upon an average, one grain of arsenic, five-sixths of a grain of oxide of copper, one grain of acid and a grain of oxide of lead, per sheet. He attributes the presence of these poisonous substances to the employment, in the manufacture of this kind of paper, of old carpet which had been dyed with Schweinfurt green, &c.—*London Lancet.*

[In the last volume of the Journal (p 68), is the notice of a case of poisoning in a child, caused by chewing a green pasteboard show-card. The green enamel on the surface of the card was found, on examination to contain arsenic.—Eds. Boston Med. & Surg. Jour.]

---

*Consumption Hospital in New York.*—The trustees of this institution have made an appeal for funds to aid them in erecting a suitable building. This appeal is headed by Peter Cooper. Drs. Alonzo Clark and John H. Griscom are among the medical names. We can hardly conceive of a more useful, a more indispensable charitable institution, in a great capital like New York, where from one-fourth to one-seventh of all the deaths are from phthisis. We sincerely hope the trustees may be successful in obtaining ample means for constructing a hospital suitable to the wants of New York.

---

The total mortality of Brooklyn, N. Y., during the year 1855, was 3,893, of which number 2,031 were males, and 1,862 females. Of this number, 424 died of consumption, 177 of pneumonia, 154 of croup, 286 of cholera infantum, 239 of scarlet fever. 48 of measles, 9 of small-pox, and 61 of typhus and typhoid fevers.—*New York Medical Times.*

